# Schimke immunoosseous dysplasia: an ultra-rare disease. a 20-year case series from the tertiary hospital in the Czech Republic

**DOI:** 10.1186/s13052-023-01413-y

**Published:** 2023-01-19

**Authors:** Jakub Zieg, Martin Bezdíčka, Michaela Němčíková, Miroslava Balaščáková, Martina Suková, Katalin Štěrbová, Karel Vondrák, Jiří Dušek, Anna Křepelová

**Affiliations:** 1 Department of Pediatrics, University Hospital Motol, Second Medical Faculty, Charles University, V Úvalu 84, 15006 Praha 5, Prague, Czech Republic; 2 Vera Vavrova Lab/VIAL, University Hospital Motol, Second Medical Faculty, Charles University, Prague, Czech Republic; 3 Department of Biology and Medical Genetics, University Hospital Motol, Second Medical Faculty, Charles University, Prague, Czech Republic; 4 Department of Pediatric Hematology and Oncology, University Hospital Motol, Second Medical Faculty, Charles University, Prague, Czech Republic; 5 Department of Pediatric Neurology, University Hospital Motol, Second Medical Faculty, Charles University, Prague, Czech Republic

**Keywords:** Schimke immunoosseous dysplasia, Case series, Nephropathy, Chronic kidney disease, Transient ischemic attacks, Transplantation

## Abstract

**Background:**

Schimke immunoosseous dysplasia (SIOD) is an ultra-rare inherited disease affecting many organ systems. Spondyloepiphyseal dysplasia, T-cell immunodeficiency and steroid resistant nephrotic syndrome are the main symptoms of this disease.

**Case presentation:**

We aimed to characterize the clinical, pathological and genetic features of SIOD patients received at tertiary Pediatric Nephrology Center, University Hospital Motol, Prague, Czech Republic during the period 2001–2021. The mean age at diagnosis was 21 months (range 18–48 months). All patients presented with growth failure, nephropathy and immunodeficiency. Infections and neurologic complications were present in most of the affected children during the course of the disease.

**Conclusions:**

Although SIOD is a disease characterized by specific features, the individual phenotype may differ. Neurologic signs can severely affect the quality of life; the view on the management of SIOD is not uniform. Currently, new therapeutic methods are required.

## Background

Schimke immunoosseous dysplasia (SIOD), first described by Schimke et al. in 1971, is an autosomal recessive multisystem disorder with prevalence of 1:1–3,000,000 live births. It is characterized by spondyloepiphyseal dysplasia causing short stature, facial dysmorphism, proteinuric nephropathy leading to progressive loss in kidney function and defective cellular immunity [1–3]. SIOD is caused by biallelic pathogenic variations in the *SMARCAL1* gene which encodes for a protein that is a member of the SWI/SNF family of proteins, involved in chromatin remodeling and the transcriptional regulation of certain genes [4]. The diagnosis is usually first suspected in a child with nephrotic proteinuria and disproportionate growth failure. Most children develop end-stage kidney disease (ESKD) before the age of 10 years. The most common kidney histologic findings are focal segmental glomerulosclerosis (FSGS) or minimal change disease [5]. There is not an effective therapy for the renal disease, so renal replacement therapy is initiated in patients with ESKD. Some authors suggest transplanting the kidney with a reduced immunosuppressive protocol due to the risk of infection in the post-transplant period [6]. Infection associated with T cell deficiency is generally the most common complication of the disease and a major cause of mortality. SIOD may be further complicated by central nervous system involvement, lung disease, enteropathy and autoimmunity. The clinical manifestation is highly variable, from rapidly progressing disease with children dying in the first years of life to milder forms with survival into adulthood. The genotype to phenotype correlation is very low, so neither the clinical course nor the outcome may be predicted [7]. This paper describes different courses of five children with this ultra-rare disease identified in our tertiary nephrology center during the period 2001 – 2021.

## Methods

Demographic characteristics, clinical and laboratory data, renal biopsy results, treatment modalities and follow-up data of SIOD patients were collected and reviewed (Tables 2 and 3). Laboratory data consisted of assessing kidney function based on serum creatinine using the Schwartz formula, blood count, serum albumin and urine analysis. The DNA samples from patients were analyzed by next generation sequencing. 48 steroid resistant nephrotic syndrome (SRNS) related genes were analyzed using Haloplex Custom Kit gene panels (Agilent Technologies, Santa Clara, CA). The libraries were prepared according to the manufacturer’s protocol and sequenced with a NextSeq 500/550 High Output Kit v2.5 (150 cycles) on a NextSeq 550 instrument (Illumina, San Diego, CA). The sequenced data were filtered and evaluated using the Varaft annotation and filter tool. The analysis revealed four distinct variants in *SMARCAL1* gene (NM_014140.4, NP_054859.2) (Table 1). The clinical significance of the identified variants was assessed using well-known prediction programs (Mutation Taster, Provean, Polyphen-2, Human Splicing Finder, UMD predictor and CADD scores). The Human Gene Mutation Database (HGMD) [8] was searched for previous descriptions of the variants, and the NCBI dbSNP for global minor allele frequencies (exclusion of common variants with a frequency of 1% or more in healthy populations found in 1000 Genomes, GnomAD and NHLBI ESP Genomes). Current standards as published by the American College of Medical Genetics and Genomics were followed to evaluate variant pathogenicity. A freely available online software tool that implements these standards was used for the evaluation of the revealed variants [9, 10]. All variants were confirmed by Sanger sequencing and the segregation of variants was verified by sequencing of parents’ DNA samples.

**Table 1:** Genetic diagnosis for our patients with SIOD

**Case**	**Detected variants** NM_014140.4 NP_054859.2	**Confirmed genotype**	**dbSNP reference number**	**HGMD number**	**ACMG evaluation**
1	c.2542G > T (p.Glu848Ter) c.1439C > T (p.Pro480Leu)	Compound heterozygous	rs119473033 rs758367100	CM020320 CM071090	Pathogenic Likely pathogenic
2	c.1382G > A (p.Gly461Asp)	Homozygous	Unknown	CM128523	Likely pathogenic
3	c.1000C > T (p.Arg334Ter) c.1382G > A (p.Gly461Asp)	Compound heterozygous	Unknown Unknown	CM071093 CM128523	Pathogenic Likely pathogenic
4	c.2542G > T (p.Glu848Ter)	Homozygous	rs119473033	CM020320	Pathogenic
5	c.2542G > T (p.Glu848Ter)	Homozygous	rs119473033	CM020320	Pathogenic

## Case presentation

The mean age at diagnosis was 21 months, interquartile range: 23.1 months and the male/female ratio was 3:2. Median time from disease manifestation to ESKD was 19.5 months.

### Case 1

**Fig. 1:**
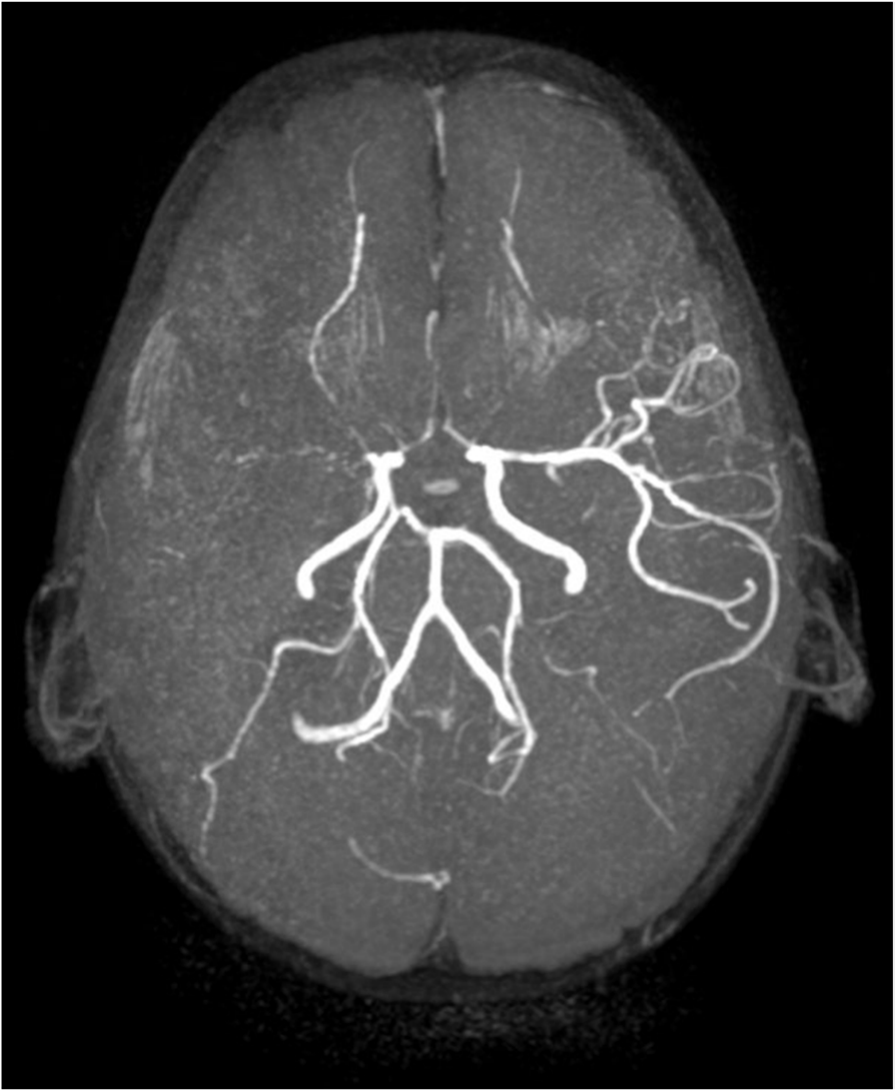
Magnetic resonance angiography imaging for case 1. The image shows bilateral stenosis of the anterior cerebral arteries and right middle cerebral artery

A three-year-old girl presented with nephrotic range proteinuria and normal kidney function. Mild transient thrombocytopenia occurred in the neonatal period. Facial dysmorphism (depressed nasal bridge and broad nasal tip), disproportionate short stature with short trunk and accentuated thoracic kyphosis and lumbar lordosis were apparent on physical examination. The course of the disease was complicated by multiple infections, including protracted *Mycoplasma pneumoniae* pneumonia, *Candida albicans* sepsis and Epstein-Barr virus infection with a prolonged febrile period. At the age of 4 years, she presented with recurrent headaches and was hospitalized with convulsions. Cerebral magnetic resonance angiography showed multiple stenoses of cerebral arteries and ischemic lesions (Fig. 1). In the following months, she had further cerebral ischemic events, which caused triplegia with expressive aphasia. Her nephrotic syndrome (NS) was initially treated with corticosteroids without response, so a renal biopsy was performed, which showed FSGS. In addition, the patient developed immune thrombocytopenia (ITP) (8 × 10^9^/L) and Coombs positive hemolytic anemia (hemoglobin 7 g/dL, reticulocytes 3.1%, LDH 7.61 μkat/L, bilirubin 11 μmol/L), leading to a diagnosis of Evans syndrome. Intravenous immunoglobulin and later immunosuppressive therapy with cyclosporine A and rituximab were ineffective and she progressed to final bone marrow failure 9 months after presentation of ITP. The patient died at the age of 5.5 years of multiorgan failure secondary to *Enterococcus cloacae* sepsis. This case was previously published [11].

### Case 2

**Fig. 2:**
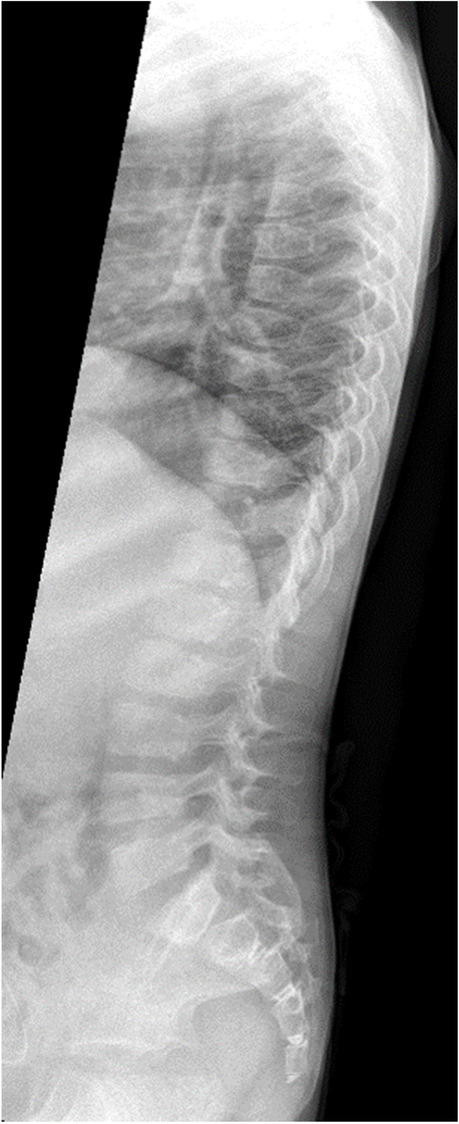
Lateral spine radiograph for case 2 showing prominent thoracic kyphosis and generalized vertebral flattening

A four-year-old boy presented with SRNS. The presenting sign, which led to the diagnosis of NS, was edema. His renal function was normal, but urine examination showed nephrotic proteinuria. Characteristic features of SIOD were present including depressed nasal bridge with broad nasal tip, disproportionate short stature with short trunk, accentuated thoracic kyphosis and lumbar lordosis (Fig. 2). Subsequently, T-cell deficiency with decreased CD4 and CD8 cells was diagnosed. He underwent kidney biopsy in our center at the age of 4.5 years, which revealed progressive focal segmental glomerulosclerosis (FSGS). He was initially treated with cyclosporin A; mycophenolate mofetil was added later. His renal function decreased rapidly and at the age of 6 years chronic peritoneal dialysis (PD) was started. The course of the disease was complicated by repeated peritoneal catheter exit site infections (caused by *Pseudomonas aeruginosa* and *Staphylococcus aureus*), which required exchange of the PD catheter and temporary transfer to hemodialysis. He also suffered from recurrent herpes zoster infections. At the last follow-up, his ESKD was managed by PD and he was administered antibiotics for exit site infection and conservative therapy of chronic kidney disease (CKD).

### Case 3

**Fig. 3:**
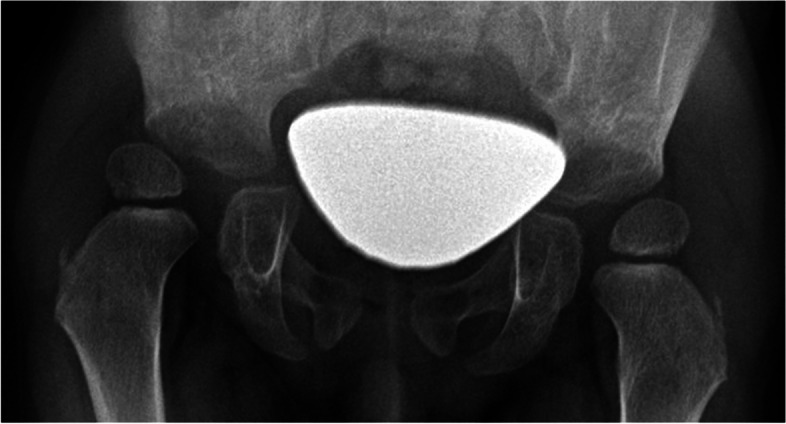
Anterioposterior hip radiograph for case 3 showing laterally displaced femoral capital epiphyses, poorly formed acetabula

**Fig. 4:**
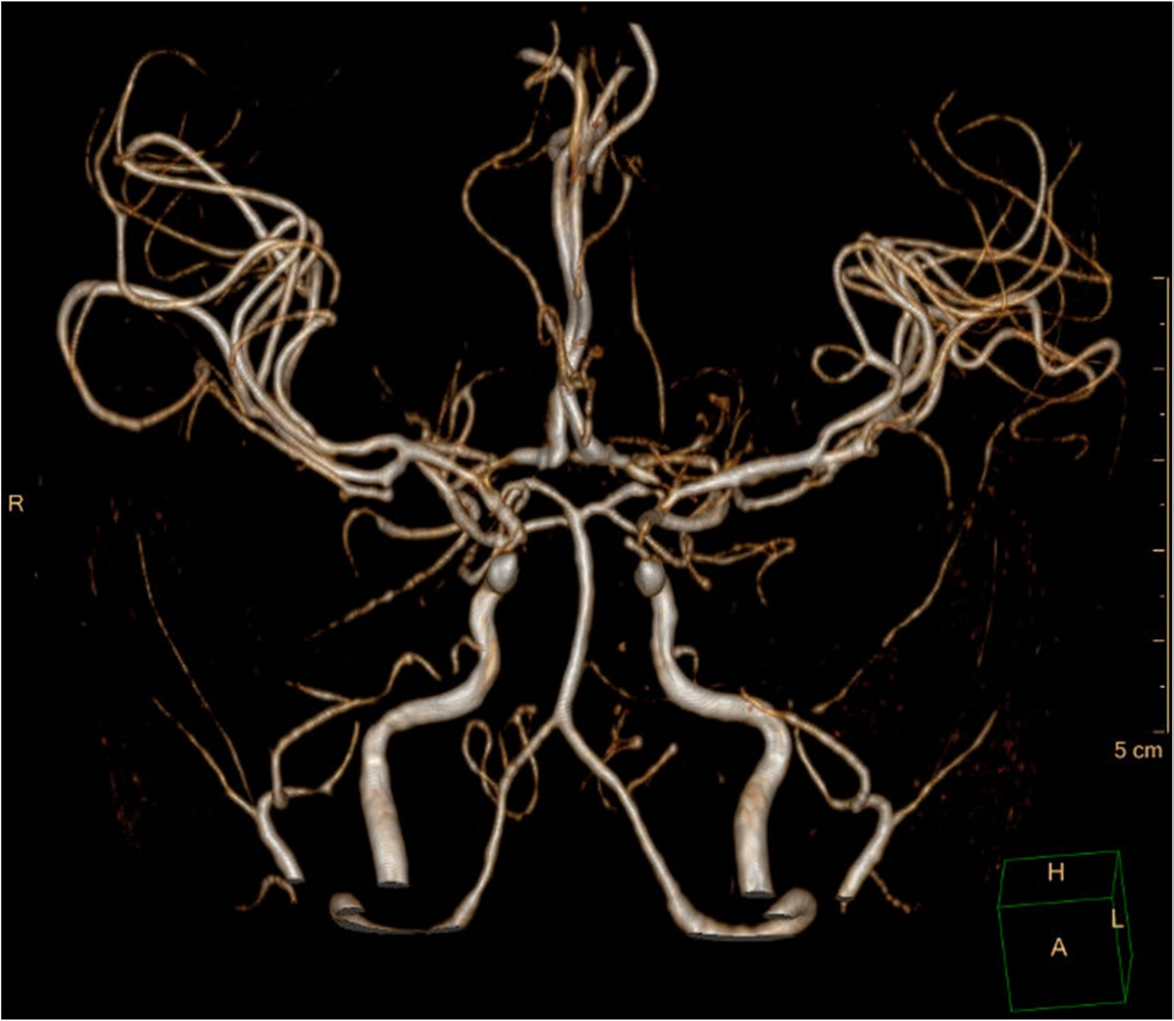
Magnetic resonance angiography for case 3. The image depicts occlusive disease filling both internal carotid arteries and both middle cerebral arteries

A 20-month-old girl was referred to our center with SRNS. She presented with eyelid edema and abdominal distension. She had been started on corticosteroid therapy for NS at the local hospital. On admission, typical facial stigmatization (low nasal bridge and broad nasal tip) and very short stature with short trunk, hyperpigmented skin macules and corneal opacities were evident. Hip radiograph showed laterally displaced femoral capital epiphyses (Fig. 3). Her kidney function was normal, urinalysis revealed nephrotic proteinuria, the blood count showed apparent leukopenia (3.5 × 10^9^/L) and immunologic examination revealed significant T-cell deficiency with decreased CD4 and CD8 cells. Typical features led us to strongly suspect a diagnosis of SIOD. Because of the rapid progression of nephropathy, PD was initiated at the age of 2.5 years. Episodic hypertension reflected insufficient compliance with fluid restriction. She started to suffer from severe neurologic complications at the age of 6 years. The most common manifestations were headaches, ischemic strokes and transient ischemic attacks, presenting as right upper limb palsy and expressive aphasia. Repeated magnetic resonance imaging (MRI) showed non-specific gliotic scars and progressive occlusive disease filling both internal carotid arteries and both middle cerebral arteries (Fig. 4). Unfortunately, over time, the occurrence of disabling neurologic symptoms was very frequent, i.e., almost daily. Repeated multidisciplinary consults recommended conservative treatment of the vascular occlusion. At the last follow-up, the patient was treated using PD and with CKD-associated complications.

### Case 4

A 25-month-old boy was referred to our center for SRNS and leukopenia. He had presented 4 months before with edema in the local hospital, where a diagnosis of NS had been made, for which corticosteroid therapy had been started. He suffered from recurrent upper respiratory infections and aphthous stomatitis. Typical features including facial stigmatization- low nasal bridge and broad nasal tip, hyperpigmented skin macules, pronounced thoracic kyphosis and short stature with short trunk pointed to a diagnosis of SIOD. His renal disease progressed to ESKD, and PD was started at the age of 3 years and 4 months. At the age of 6 years, he was admitted to the local hospital with convulsions and hypertension. Brain MRI showed ischemic lesions in the area supplied by the right middle cerebral artery. Frequent migraine-like headaches appeared later. He suffered from headaches and transient ischemic attacks lasting for up to 20 h as well as right upper limb palsy 6 months after the previous episode. At the last follow-up, our patient was treated with PD and conservative therapy for CKD.

### Case 5

An eighteen-month-old boy presented with lower extremity edema and gradually fulfilled the diagnosis of NS. He did not respond to steroid treatment and was classified as SRNS. Kidney biopsy performed at the local hospital 2 months after the onset of the disease showed IgM nephropathy with segmental sclerotization. Upon admission to our center, he had mild edema of his lower extremities, normal kidney function and nephrotic proteinuria with normal serum albumin. Characteristic features of SIOD including low nasal bridge and broad nasal tip, disproportionate short stature with short trunk, accentuated thoracic kyphosis, hyperpigmented skin macules and corneal opacities were present along with laboratory finding of leukopenia and T- cell deficiency. He had protracted adenovirus gastroenteritis at the age of 2 years. After 3 months, he suffered from acute COVID-19 infection with NS decompensation, which required increased diuretic therapy and infusions of 20% human albumin solution. At the age of 3.3 years he reached ESKD and PD was started. At the last follow-up, he was in satisfactory clinical condition treated by dialysis and CKD conservative therapy.

Tables 2, 3, 4 and 5 summarize clinical, laboratory and auxological parameters for our patients.

**Table 2:** Clinical features for our patients at the time of diagnosis

Case	Facial features	Edemas	Skin manifestations	Growth	Ophtalmological features	Dental anomalies	Nephropathy	CKD stage
1	Depressed nasal bridge, broad nasal tip	No	Hyperpigmented macules	Disproportionate short stature	No	No	SRNS	1
2	Depressed nasal bridge, broad nasal tip	Yes	No	Disproportionate short stature	No	No	SRNS	1
3	Depressed nasal bridge, broad nasal tip	Yes	Hyperpigmented macules	Disproportionate short stature	Corneal opacities	Microdontia	SRNS	1
4	Depressed nasal bridge, broad nasal tip	Yes	Hyperpigmented macules	Disproportionate short stature	No	Microdontia	SRNS	1
5	Depressed nasal bridge, broad nasal tip	Yes	Hyperpigmented macules	Disproportionate short stature	Corneal opacities	Microdontia	SRNS	1

**Table 3:** Laboratory parameters for our patients at the time of diagnosis

Case	Age (months)	Sex	WBC X10^9^/L (5–17 × 10^9^/L)	HGB (g/L) (11–13.7 g/L)	PLT X10^9^/L (250–450 × 10^9^/L)	GFR (mL/min/1.73m^2^)	Serum albumin (g/L) (35 – 55 g/L)
1	36	F	4.9	138	415	100	38
2	48	M	2.6	97	437	> 90	N/A
3	19	F	3.4	124	349	> 90	20.4
4	21	M	4.4	139	388	> 90	22.6
5	18	M	4.1	88	835	> 90	39.7
	**TSH (mIU/L)**	**Protein/creatinine ratio (mg/mmol)**	**IgG (g/L)**	**CD4 (× 10**^**9**^**/L) (0.9 – 2.9)**	**CD8 (× 10**^**9**^**/L) (0.345–0.42)**	**CD4/CD8 (× 10**^**9**^**/L) (1.0–3.0)**	**TSH (mIU/L) (0.7–6.6)**
1	6.5	850 mg/m^2^	1.63	0.02	0.03	0.5	6.5
2	3.52	NA	6.33	0.19	0.14	1.3	3.52
3	12.13	4362 mg/mmol	1.31	0.27	0.08	3.5	12.13
4	9.7	1656 mg/mmol	0.89	0.41	0.47	0.9	9.7
5	2.81	311 mg/mmol	1.05	0.30	0.18	1.7	2.81

*NA* Not available

**Table 4:** Clinical and laboratory parameters for our patients at last follow-up

Case	Age at last follow-up	Height at last follow-up (cm, percentile)	Hematologic symptoms	CKD Stage at last follow-up	Age at ESKD (years)	Dialysis	Infection complications	Neurologic complications	Autoimmune diseases
1	5 y 8 m^a^	78, < 3^rd^	Leucopenia WBC 2.2 × 10^9^/L, Evans syndrome, bone marrow failure	1	-	No	Mycoplasma pneumoniae pneumonia, Candida albicans sepsis, protracted EBV systemic infection, Enterococcus cloacae sepsis	Recurrent headaches, convulsions, cerebral ischemic events, triplegia with expressive aphasia	Yes
2	17 y 9 m	123, < 3^rd^	Leucopenia WBC 3.4 × 10^9^/L	5	6	Yes	Recurrent herpes zoster skin infection, repeated peritoneal catheter exit site *Pseudomonas aeruginosa* and *Staphylococccus aureus* infections	No	No
3	7 y 3 m	80, < 3^rd^	Leucopenia WBC 3.0 × 10^9^/L	5	2.5	Yes	No	Recurrent headaches, transient ischemic attacks presenting as right upper limb palsy and expressive aphasia	No
4	7y	86, < 3^rd^	Leucopenia WBC 2.4 × 10^9^/L	5	3.3	Yes	Recurrent upper respiratory infections, aphthous stomatitis	Recurrent headaches, transient ischemic attacks, right upper limb palsy	No
5	2 y 6 m	74, < 3^rd^	Leucopenia WBC 3.4 × 10^9^/L	5	3.3	Yes	Severe course of COVID-19 infection	No	No

^a^Patient died at this age

**Table 5:** Auxological parameters for our patients

Case	Week of gestation	BW/BL (BW percentile)	Age of assess-ment (years)	Weight Height	Head circumference	Chest circumference	Arm span/height ratio	Sitting height
1	30	1450 g/39 cm (< 3^rd^)	4.2	9 kg (-6 SD), 76 cm (-6.8 SD)	44 cm (-4.2 SD)	48 cm (-3.6 SD)	1	NA
2	35	1190 g/31 cm (< 3^rd^)	16	18.6 kg (-8.3 SD), 121.4 cm (-7.4 SD)	52.3 cm (-2.3 SD)	63.6 cm (-3.2 SD)	NA	53.6 cm (-5.9 SD)
3	34	1430 g/42 cm (< 3^rd^)	4.5	8.6 kg (-6.8 SD), 80.2 cm (-6.3 SD)	47.2 cm (-2.1 SD)	48.1 cm (-1.9 SD)	1.02	NA
4	36	1780 g/41 cm (< 3^rd^)	4	11.7 kg (-3.6 SD), 82.6 cm (-5.4 SD)	48.8 cm (-1.4 SD)	51.5 cm (-1.1 SD)	1.1	46.8 cm (-5.2 SD)
5	35	2000 g/44 cm (8^th^)	3.7	9 kg (-6.1SD), 74.5 cm (-7.1SD)	45.8 cm (-3.4SD)	50 cm (-1.7 SD)	1.02	41 cm (-4.9 SD)

*BW* Birth weight, *BL* Birth length, *NA* Not available, *SD* Standard deviation

## Discussion and conclusions

### Signs of the disease and complications

Our cases confirm that SIOD is characterized by low renal and high extrarenal phenotypic variability. Short stature and proteinuric nephropathy are the typical hallmarks of SIOD and usually the first signs suggesting the disease. [5] All our patients were identified initially by nephrologists involved in the diagnosis of NS, which represents a very heterogeneous group of diseases. Determining the etiology of SRNS is crucial for the proper management of these patients. The proportion of genetic SRNS from all SRNS patients has been reported in 14–64% cases; our national study showed that 38% of children with SRNS have a confirmed genetic etiology [12]. Some of these children present with syndromic NS with typical specific extrarenal features that allow clinicians to establish an accurate diagnosis.

All children were born preterm with low birth weight, intrauterine growth retardation (IUGR), characteristic facial stigmatization (broad nasal bridge) and the majority of them had pigmented skin macules. Edema was the presenting sign in four children, and two presented with only nephrotic proteinuria. Renal biopsy was performed in three children; FSGS was found in two cases and IgM nephropathy was revealed in one child. Interestingly, based on our knowledge, our case is the first IgM nephropathy diagnosed in an SIOD patient reported to date. Generally, IgM nephropathy is an immune complex-mediated glomerulopathy characterized by diffuse mesangial immunoglobulin M deposits. Patients with IgM nephropathy are more often dependent on or resistant to corticosteroids. [13] However, histologic diagnosis is not decisive for the management of SIOD, so it may be avoided. Therefore, kidney histology results from many SIOD children are unavailable. Of note, four children from our cohort (80%) reached ESKD due to chronic progressive proteinuric nephropathy at the age ≤ 6 years.

The main complications and the major cause of mortality of SIOD patients are infections mainly associated with T-cell deficiency. Four patients from our cohort suffered from recurrent or protracted infections. We tried to identify the etiology of the infections and we treated them appropriately. Severe sepsis was the cause of death in case 1; moreover, we observed significant decompensation of the NS with generalized edema requiring albumin infusion and intensification of diuretic therapy during a COVID-19 infection in case 5.

Disproportionate short stature with short trunk is the result of spondyloepiphyseal dysplasia. An anthropometric study by Lücke et al. found that SIOD patients differed significantly from other individuals with CKD. The ratio of sitting height:leg length < 0.83 was specific for SIOD [14].

*SMARCAL*1 deficiency may lead to vascular brain disease; the resulting neurologic symptoms may be severe and can significantly reduce the quality of life. In some patients, the brain damage may be irreversible. Transient ischemic attacks (TIA) were first described by Ehrich et al. in three children who had perfusion defects revealed by positron emission tomography [15]. The expression of *SMARCAL1* in brain tissue and the association of SMARCAL1 deficiency and abnormal brain development was reported later [16]. Kilic et al. hypothesized that *SMARCAL1* may regulate the expression of genes necessary for neurological homeostasis. In addition, they postulated that disturbed expression of *SMARCAL1* in the vasculature and smooth muscle may contribute to vascular dysfunction in SIOD [17]. Based on our current knowledge, reversible cerebral vasoconstriction syndrome, atherosclerosis and reduced elastogenesis contribute to the development of cerebral signs [18, 19]. The trigger of cerebral attacks may be blood pressure fluctuations, but these were not always present in our patients. Endothelial injury is also accentuated by CKD complications in SIOD individuals. In addition, management of hypertension in children treated with dialysis is challenging. We observed triplegia with expressive aphasia as a result of recurrent ischemic attacks in case 1. Cases 3 and 4 suffered from recurrent transient ischemic attacks, strokes and headaches. With time, the duration of attacks became longer, and symptoms were more pronounced. All our patients were treated by antiaggregation; we tried administering calcium channel blockers, minoxidil, beta blockers, paracetamol and non-steroidal anti-inflammatory drugs for neurologic symptoms with unsatisfactory effects. Unfortunately, no potent therapy for these complications has been established yet. Invasive intravascular treatment and thrombolysis are not recommended because of the age, body weight, abnormal tissue of the vessels and frequent recurrency of episodes.

Hematologic disease may occur during the course of SIOD disease. Leukopenia is a characteristic sign of the disease and was present in all our reported children. Evans syndrome resistant to multiple therapy including rituximab and bone marrow failure developed in case 1 [11]. In children with preserved diuresis, nephrotic proteinuria results in severe hypogammaglobulinemia, which aggravates the immunodeficient state in SIOD.

### Genotype–phenotype correlation

Homozygous or compound heterozygous causal variants were identified in all our patients using next generation sequencing. SIOD is a disease with a variable phenotypic presentation. While spondyloepiphyseal dysplasia, dysmorphic features, renal disease and T-cell deficiency are typical features of SIOD, neurologic symptoms, autoimmune diseases and other organ symptoms are present in only a proportion of affected individuals. The pathogenic mechanisms leading to SIOD are not yet fully understood. Finding modifiers and other genes associated with SIOD is expected [20]. The phenotype varies from mild to severe disease; interestingly, the genotype to phenotype correlation is very weak, probably due to variable gene expression, oligogenic inheritance, epigenetics and environmental factors [3]. Lama et al. reported three siblings with different disease courses. While one boy died with severe nephropathy on hemodialysis at the age of 12 years, his brother survived into adulthood. He was started on hemodialysis when he was 22 years old and subsequently received a kidney graft. Their sister maintained normal kidney function throughout childhood [21].

### Management

To date, no effective causal therapy for SIOD has been found. It is important to make the correct diagnosis early. Our reported children were diagnosed late and were exposed to unnecessary immunosuppressive therapy, which could facilitate the development of life-threatening infections. Nephropathy in SIOD is not amendable to immunosuppressive therapy. Steroids for manifestations of NS were used in all our patients before the correct diagnosis was established. Based on current knowledge, this therapy is of no value for genetic SRNS. Angiotensin-converting enzyme inhibitors may decrease proteinuria in these individuals; however, this has not been demonstrated by studies yet. Four patients from our cohort reached ESKD and PD was started. Lücke et al. published a case series of children with SIOD who successfully underwent kidney transplantation (Tx) with reduced immunosuppression due to T-cell immunodeficiency [6]. However, generally, there is a reluctance to perform kidney Tx because of the high risk of severe infections. On the other hand, long-term dialysis poses a threat of systemic side effects including progression of atherosclerosis, cardiovascular disease precipitating central nervous system disease and shortened life span. SIOD is and ultra-rare disease and clinical course of these individuals after kidney Tx could not have been studied well yet. [22] There is only limited evidence supporting kidney Tx in these children. Therefore, performing kidney Tx remains controversial in SIOD patients mainly due to the risk of life-threatening infections. Importantly, the majority of our patients have serious extrarenal symptoms, which may progress regardless of kidney Tx.

A special vaccination protocol for children with T-cell deficiency with the avoidance of live-attenuated vaccines was followed. Antibiotic prophylaxis (cotrimoxazole) was prescribed for SIOD patients to prevent opportunistic infection. [3] Although previous reports on bone marrow Tx have been rather disappointing, as four of five patients in the cohort did not survive [23], promising results have been published recently by Bertaina et al., who performed alpha/beta T-cell depleted haploidentical stem cell Tx followed by kidney Tx from a living donor. This revolutionary method lessens the risk of rejection and enables the minimization of immunosuppression with subsequent discontinuation of immunosuppressive medication. More experience with this promising therapeutic method is expected [24].

### Limitations

The limitation of our study is the small sample size, but SIOD is an extremely rare disease and our study reports all the children presenting with this disease at our center over a period of 20 years.

## Conclusion

In conclusion, we report our tertiary center cohort of five children with SIOD assessed over a 20-year period of time. To our knowledge, this is the largest single center cohort of this very rare disease reported to date. Pediatricians should be aware of this disease associated with a typical phenotype. Identification of SIOD is of utmost importance for further therapy. Management of SIOD is challenging, as the majority of affected individuals suffer from severe complications limiting their quality of life and longevity. New therapeutic approaches are needed to improve the outcome of SIOD.

## Data Availability

Data sharing not applicable to this article as no datasets were generated or analyzed during the current study.

## Abbreviations

CKD: chronic kidney disease
ESKD: end-stage kidney disease
FSGS: focal segmental glomeruslosclerosis
ITP: immune thrombocytopenia
IUGR: intrauterine growth retardation
MRI: magnetic resonance imaging
NS: nephrotic syndrome
PD: peritoneal dialysis
SIOD: Schimke immunoosseous dysplasia
Tx: transplantation
SRNS: steroid resistant nephrotic syndrome
